# Overexpression of CRABP2 inhibits dexamethasone-induced apoptosis in human osteoblast cells

**DOI:** 10.1186/s13018-021-02386-6

**Published:** 2021-04-20

**Authors:** Haiping Zhang, Ziliang Yu, Farui Sun, Jin Jin

**Affiliations:** 1 Department of Orthopaedics, Affiliated Hospital 2 of Nantong University, Nantong University, Nantong, 226000 Jiangsu People’s Republic of China; 2 Department of Orthopaedics, Huangshi Central Hospital (Affiliated Hospital of Hubei Polytechnic University), Edong Healthcare Group, Huangshi, 435000 Hubei People’s Republic of China; 3 Medical College, Wuhan University of Science and Technology, Wuhan, China; 4 Department of Endocrinology, the Affiliated Hospital of Xuzhou medical University, Xuzhou, 221000 China

**Keywords:** CRABP2, Bioinformatic analysis, Apoptosis, Osteonecrosis of the femoral head

## Abstract

**Background:**

The purpose of the current study was to explore the role and underlying mechanism of cellular retinoic acid binding protein 2 (CRABP2) in dexamethasone (DEX)-induced apoptosis in human osteoblast cells.

**Methods:**

GSE10311 was downloaded from the Gene Expression Omnibus (GEO) database to identify the differentially expressed genes (DEGs) by the limma/R package. Primary human osteoblast was isolated and treated with different concentration of DEX (0, 10^-8^, 10^-7^, 10^-6^, 10^-5^, and 10^-4^ mol/L), and cell viability and flow cytometry were used to detect cell proliferation and apoptosis. A CRABP2 overexpression plasmid (oe-CRABP2) was used to overexpress CRABP2, and western blotting was conducted to detect protein expression.

**Results:**

We found that CRABP2 was downregulated in the DEX-treated group. Kyoto Encyclopedia of Genes and Genomes (KEGG) pathway analyses indicated that DEGs were associated with PI3K/Akt signaling pathway. DEX downregulated CRABP2 gene and protein expression, inhibited viability, and induced human osteoblast apoptosis. Overexpression of CRABP2 reversed DEX-induced apoptosis in human osteoblast. Moreover, overexpression of CRABP2 delayed the progression of DEX-induced osteonecrosis of the femoral head (ONFH) animal model.

**Conclusion:**

In conclusion, CRABP2 is effective at inhibiting DEX-induced human osteoblast apoptosis and delayed ONFH progression.

## Introduction

Glucocorticoids are important drugs in the treatment of many inflammatory, autoimmune, allergic diseases, cancer, and organ transplantations in humans and animals [1–4]. Furthermore, the use of high-dose corticosteroids can lead to irreversible adverse effects, such as osteonecrosis of the femoral head (ONFH) [5, 6]. The progression of patients with ONFH with an insidious onset usually deteriorated further and eventually led to joint collapse and hip osteoarthritis [7–9]. Total hip arthroplasty (THA) is the terminal treatment of ONFH [10]. However, the effect of THA treatment is often not satisfactory and with immense financial burden for patients [11, 12]. Therefore, in order to develop effective therapies for ONFH treatment, a better understanding of the molecular mechanisms underlying the ONFH pathogenesis is warranted [13].

There are plenty of public free databases, such as the Cancer Genome Atlas (TCGA) and the Gene Expression Omnibus (GEO), from which abundant information can be excavated via various bioinformatics methods [14]. In this study, we revealed that CRABP2 was differentially expressed between dexamethasone and control groups in GSE10311. Cellular retinoic acid-binding proteins, CRABP1 and CRABP2, are small cytosolic proteins that belong to a family of two isotypes. CRABP2 is correlated with poor prognosis of multiple cancers. Wu et al. [15] found that CRABP2 promotes metastasis of lung cancer cells via HuR and Integrin β1/FAK/ERK signaling pathway. Chen et al. [16] revealed that downregulation of CRABP2 inhibit the tumorigenesis of hepatocellular carcinoma in vivo and in vitro. However, the role of CRABP2 in regulating dexamethasone induced osteoblast apoptosis and ONFH was unknown.

In this study, GSE10311 was downloaded and performed bioinformatic analysis to identify the differentially expressed genes under dexamethasone treatment in osteoblast. A ONFH rat model was created to investigate the treatment role of CRABP2 in ONFH.

## Materials and methods

### Microarray analysis

The mRNA expression profile GSE10311 was retrieved from the GEO database (https://www.ncbi.nlm.nih.gov/geo/). In this microarray, 3 samples of primary human osteoblasts were treated with dexamethasone (DEX, 10^-4^ mol/L) for 24 h and 6 samples without treatment as controls. Differentially expressed genes (DEX-treated vs Con) were further analyzed by the R package “Limma” from the Bioconductor project [17]. |logFC| > 1 and *P* value < 0.05 was set as the cutoff point.

### Function enrichment analysis

Gene Ontology (GO) analysis, which includes biological processes (BP), cellular components (CC) and molecular function (MF), and Kyoto Encyclopedia of Gene and Genomes (KEGG) pathway enrichment analysis were carried out using the clusterProfiler (version 3.10.1) package [18]. The identified differentially expressed genes were inputted into the STRING database to obtain the protein-protein interaction network (http://string.embl.de/). Subnetwork models were selected using the plugin molecular complex detection (MCODE) application in Cytoscape, with the following criteria: degree cutoff = 2, node score cutoff = 0.2, k-core = 2, and max depth = 100 [19].

### Cell culture and plasmid transfection

The human osteoblast was isolated and cultured as previously described. In brief, from the American Type Culture Collection (ATCC, CRL-2593) and maintained in DMEM (Gibco, Life Technologies, Carlsbad, CA, USA) supplemented with 10% FBS (Gibco, Life Technologies, Carlsbad, CA, USA), 10 mM HEPES (Sigma Aldrich, Poole, UK), and 0.1% penicillin-streptomycin (Sigma Aldrich, Poole, UK). The CRABP2-pcDNA3 plasmid (oe-CRABP2) was synthesized by Santa Cruz and transfected following the manufacturer’s protocol.

Then, human osteoblasts were plated onto the cover glass of a confocal Petri dish (NEST, Hong Kong, China) for transient transfection. DMEM (1 mL) containing 10% FBS was added to the dish, and the cells were cultured for 24 h prior to transfection. Transfection was performed using Lipofectamine 3000 (Thermo Fisher Scientific) and Opti-MEM reduced-serum media (Life Technologies, Waltham, Massachusetts, USA) according to the manufacturer’s instructions. To explore the mechanisms of CRABP2-mediated osteoblast apoptosis, the PI3K inhibitor LY294002 was used to pretreat cells (20 μM, MCE, Shanghai, China) for 2 h followed by stimulation with CRABP2-pcDNA3 for 12 h. The choice of inhibitor concentrations and time course was based on a previous study [20].

### Cell viability assay

The cytotoxicity effect of DEX in osteoblast was evaluated by MTT assay (Solarbio, Beijing, China). Previous study showed that DEX at 10^-4^ M had obviously effects on the apoptosis of osteoblast. In the present study, cells in a 96-well plate (5×10^3^ cells/well) were treated with different concentrations of DEX (10^-8^, 10^-7^, 10^-6^, 10^-5^, and 10^-4^ M) for 24 h. MTT assay was performed as described previously [21].

### Apoptosis flow cytometry assay

Flow cytometry was utilized to quantify cell apoptosis in osteoblast using Annexin V-FITC/PI Kit (Hanbio, Shanghai, China) following the indicated treatment. In brief, the cells were cultured in 12-well plate and were trypsinized and stained with PI-conjugated anti-Annexin V antibodies under darkness for 20 min at room temperature. Next, they were analyzed by flow cytometry using Accuri C6 plus (Becton Dickinson, Franklin Lakes, NJ, USA).

### Western blot analysis

The cells were extracted with lysis buffer containing 50 mM Tris-Cl (pH 7.4), 150 mM NaCl, 1 mM EDTA, 1 mM EGTA, 10 μg/ml aprotinin, 10 μg/ml leupeptin, 5 mM phenylmethylsulfonyl fluoride, 1 mM DTT, and 1% Triton X-100. The proteins were transferred to polyvinylidene difluoride (PVDF) membranes. After blocking with 5% non-fat milk, the PVDF membranes were incubated with primary antibodies in blocking buffer overnight at 4°C, followed with the appropriate secondary antibodies for 2 h at room temperature. The filter was incubated with the electroencephalographic substrate and exposed to X-ray film (Kodak).

### Animal studies

This study was performed according to the Ethics Committee of XXX Hospital. Twenty-four 8-week-old female Sprague-Dawley rats weighing 320 ± 30 g were used. The rats were randomly and equally subdivided into five groups: (1) a control group (rats treated with PBS), (2) methylprednisolone (MPS, ONFH) group, (3) ONFH + CRABP2-pcDNA3 group, and (4) ONFH+ ONFH + CRABP2-pcDNA3+LY294002. To induce ONFH in rats, pulsed high-dose 100 mg/(kg•d) MPS was intramuscularly injected within the first week and sustained low-dose 21 mg/(kg•d) MPS was administered after the next 4 weeks. Five weeks later, the rats were euthanized, and the femoral heads were examined by HE and Masson analysis.

### Immunohistochemistry (IHC)

The calvarial were decalcified, embedded in paraffin and sectioned (5 μm). Afterward, for immunohistochemical assessment of the osteogenesis, the marker staining of COL1A was observed. Sections were incubated 12 h at 4°C with COL1A (1:500; Abcam). Afterward, the sections were incubated with the biotinylated secondary antibodies (Zhongshan Biotechnology Corporation, Ltd., China).

### Statistical analysis

All data are presented as the means ± SD and analysis by SPSS19.0 software (SPSS, Chicago, IL). Differences among different groups were calculated with one-way analysis of variance (ANOVA) and post-analysis by Tukey’s honestly significant difference test. *P* < 0.05 was employed to indicate statistical significance.

## Results

### Identification of differentially expressed genes

**Fig. 1:**
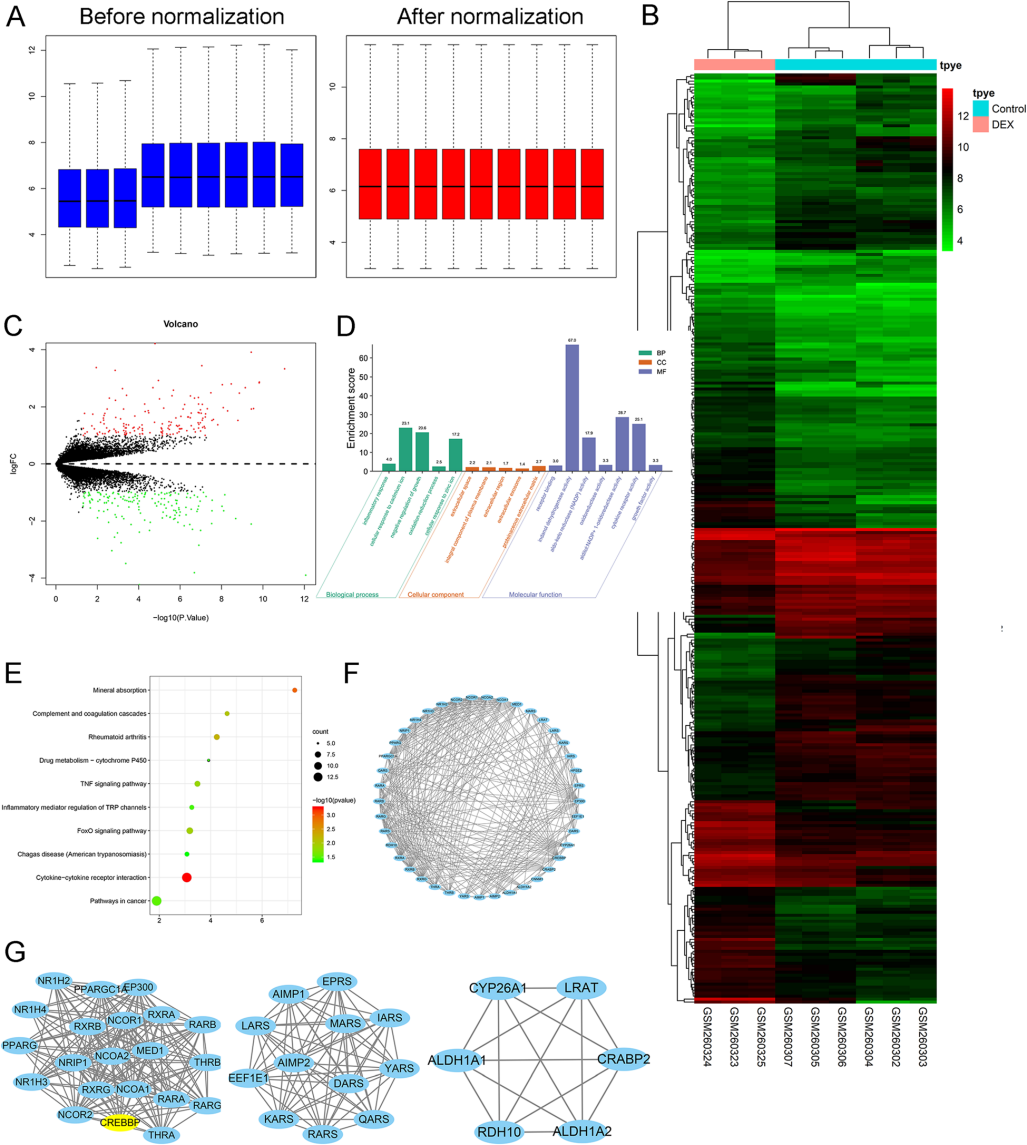
Identification of DEGs in human osteoblasts exposed to DEX. **a** Comparison of expression value between before normalization and after normalization. **b** Heatmap of expression of differentially-expressed genes: DEX vs. control. **c** Volcano plot of differentially expressed genes between DEX and control groups. The *x*-axis represents the -log10 (*P* value), and the *y*-axis represents the logFC. **d** Gene ontology of the differentially expressed genes. The *x*-axis represents the enriched gene ontology terms, and the *y*-axis represents the enrichment score. **e** Top ten KEGG terms for the differentially expressed mRNA. The abscissa represents the enriched score, and the ordinate represents the KEGG pathway terms. *P* values indicate larger values from blue through to red, and a larger node size represents a higher number of enriched genes. **f** protein-protein interaction of the differentially expressed genes. **g** Identification of a top 3 sub-network using MCODE in Cytoscape software

The box plot of the log expression values for all genes in each sample before and after normalization was drawn (Fig. 1a). The median values of each sample were extremely similar, indicating that the data should be further analyzed. A heatmap of the identified DEGs is shown in Fig. 1b and a volcano plot of the DEGs is shown in Fig. 1c. A total of 311 DEGs were identified, among which 158 DEGs were downregulated, while 153 DEGs were upregulated.

Among the 311 DEGs, CRABP2 was selected for its most significant changes (criteria of LogFC >4 and *P* < .001) for further study.

All DEGs were imported to DAVID software, and GO analysis results demonstrated that upregulated and downregulated DEGs were particularly enriched in the following biological processes (BP): Inflammatory response, cellular response to cadmium ion, negative regulation of growth, oxidation-reduction process, cellular response to zinc ion, cellular component (CC): extracellular space, integral component of plasma membrane, extracellular region, extracellular exosome, proteinaceous extracellular matrix, and molecular function (MF): receptor binding, indanol dehydrogenase activity, aldo-keto reductase (NADP) activity, oxidoreductase activity, alditol:NADP+ 1-oxidoreductase activity, cytokine receptor activity, and growth factor activity (Fig. 1d). The KEGG pathway analysis revealed that the DEGs were significantly enriched in mineral absorption, complement coagulation cascades, rheumatoid arthritis, drug metabolism-cytochrome P450, TNF signaling pathway, inflammatory mediator regulation of TRP channels, FoxO signaling pathway, Chagas disease, cytokine-cytokine receptor interaction, and pathway in cancer (Fig. 1e). The final network for the protein-protein interaction is composed of 158 nodes and 655 edges (Fig. 1f). We used the MCODE plug-in to analyze the PPI network of common DEGs for functional modules and core genes. CRABP2 was identified the core gene and was further analyzed in this study (Fig. 1g).

### DEX promotes apoptosis of osteoblast

**Fig. 2:**
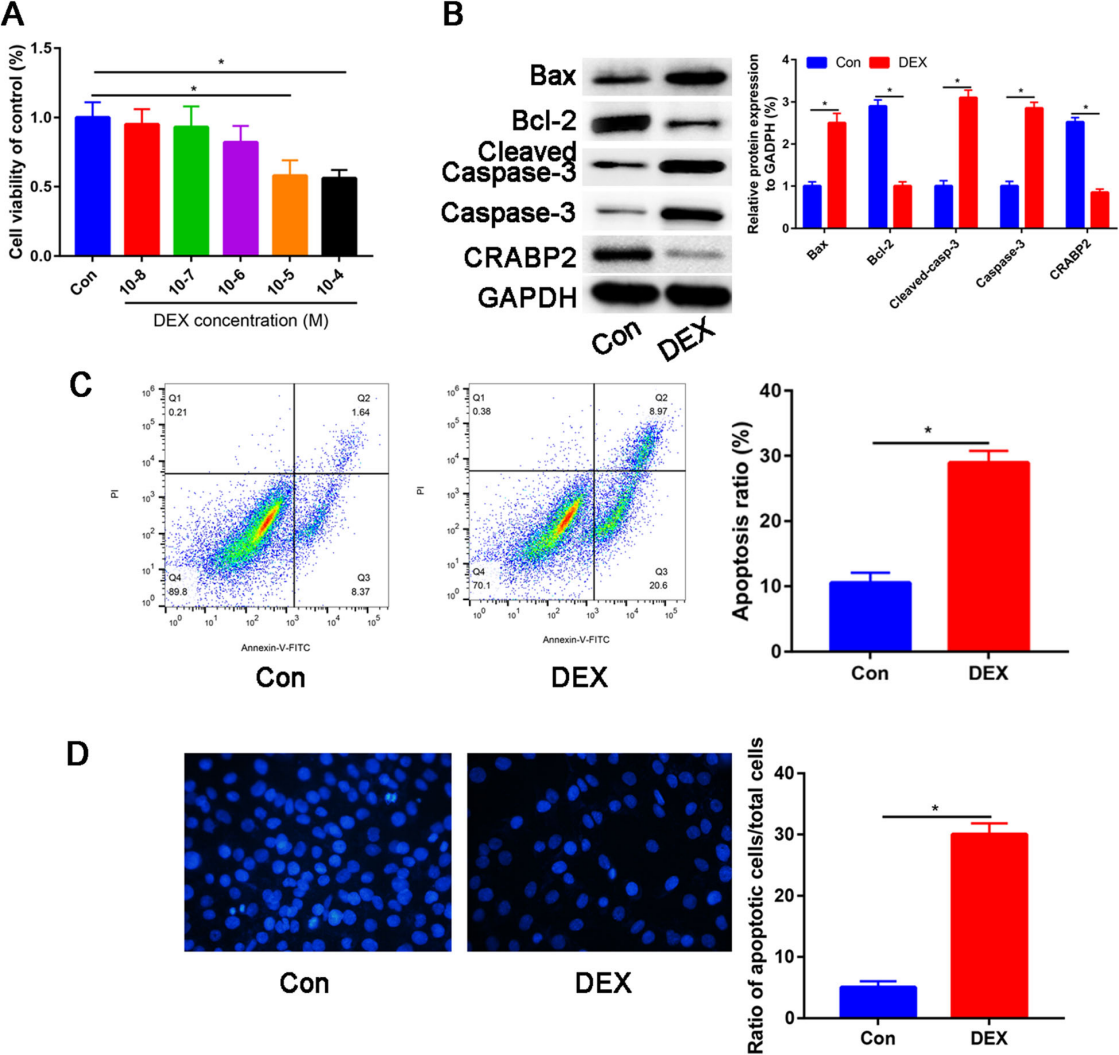
The apoptosis assay revealed that DEX promoted cell apoptosis in osteoblast. **a** Effects of DEX on osteoblast proliferation, as demonstrated by MTT assay. **b** Protein levels of Bax, Bcl-2, Cleaved caspase-3, Caspase-3, and CRABP2 were detected by western blotting after DEX treatment. **c** Flow cytometry after Annexin-V/PI staining and quantitative analysis of the apoptosis ratio after DEX treatment. **d** Apoptosis by DAPI staining after DEX treatment (×100 magnification); *P<0.05, DEX: dexamethasone

As illustrated in Fig. 2a, the cell viability of osteoblast was significantly inhibited by DEX in a dose-dependent manner (*P*<0.05), and at 10^-4^ M DEX, the cell viability reached 48% of that of the control group (Fig. 2a).

Furthermore, we assessed the CRABP2 protein expression between control and DEX groups (Fig. 2b). The CRABP2 protein expression was significantly downregulated after DEX treatment. Moreover, proapoptotic proteins (Bax, Cleaved caspase-3, and Caspase3) were significantly upregulated after DEX treatment. And antiapoptotic protein (Bcl-2) was downregulated after DEX treatment (Fig. 2b). Apoptosis was detected via Annexin V/PI double staining and DAPI staining. The apoptosis ratio was increased by 30.5% (Fig. 2c), which indicated that exposure of osteoblast to 10^-4^ M DEX significantly increased cell apoptosis. Through DAPI staining, we observed that DEX increases the number of apoptotic cells (Fig. 2d).

### Overexpression of CRABP2 rescued DEX-induced apoptosis in osteoblast

**Fig. 3:**
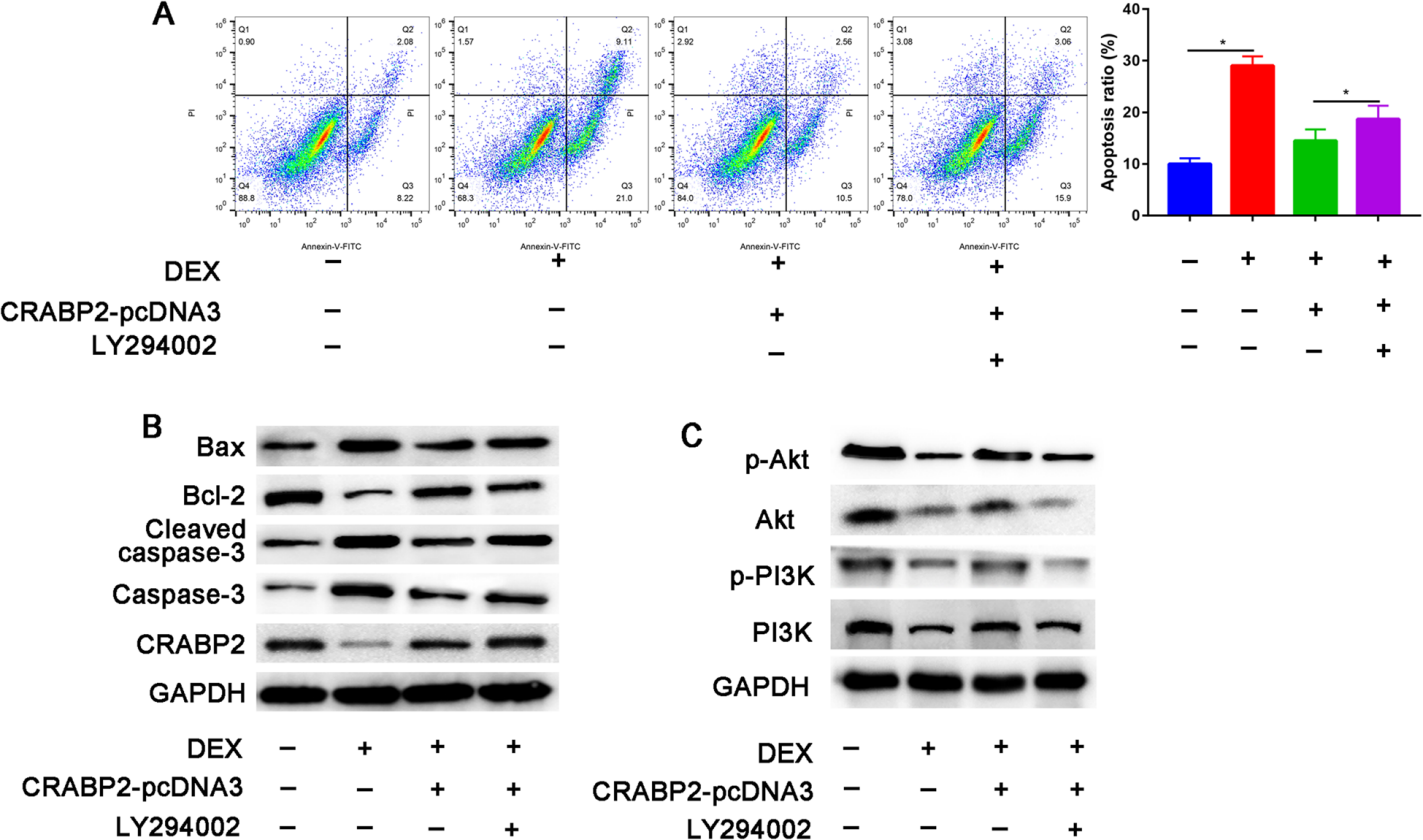
The effects of CRABP2 overexpression on DEX-induced apoptosis of osteoblast. Osteoblasts were pretreated with a CRABP2 expression plasmid and/or PI3K inhibitor (LY294002) 12 h before DEX treatment. **a** Flow cytometry after Annexin-V/PI staining analysis of apoptosis after DEX (10^-6^ M, 24 h), DEX+ CRABP2 pcDNA3, and DEX+ CRABP2 pcDNA3+LY294002 treatments. **b** Western blotting was performed to analyze the expression levels of Bax, Bcl-2, cleaved-Caspase-3, Caspase-3, CRABP2 **c**, PI3K, p-PI3K, AKT, and p-AKT in osteoblast after DEX (10^-6^ M, 24 h), DEX+ CRABP2 pcDNA3, and DEX+ CRABP2 pcDNA3+LY294002 treatments. *P<0.05, DEX: dexamethasone

As shown in Fig. 3a, compared with the control group, Dex exposure can significantly induce the occurrence of apoptotic response, while CRABP2 overexpression pretreatment can reverse the above changes (all *P* < 0.05).

The upregulation of apoptosis-related proteins (Bax, Cleaved caspase-3, and Caspase3) induced by DEX was reversed by CRABP2-pcDNA3, as shown by western blot analysis, while a marked increase in Bcl-2, an apoptosis-related protein, was observed in CRABP2-pcDNA3 compared with DEX using western blot analysis (Fig. 3b).

It is evident from Fig. 3c, PI3K and Akt activation was significantly reduced after incubation with DEX, and CRABP2 overexpression partially restored both PI3K and Akt phosphorylation. Pretreatment with LY294002 of DEX and CRABP2-pcDNA3-treated osteoblast significantly decreased the expression of p-PI3K and p-Akt compared with administration of DEX and CRABP2-pcDNA3 alone. These data suggested that CRABP2 restored the activation level of the PI3K/Akt signaling cascades blocked by LY294002 in osteoblast.

### Overexpression of CRABP2 delayed progression of OFNH in animal model

**Fig. 4:**
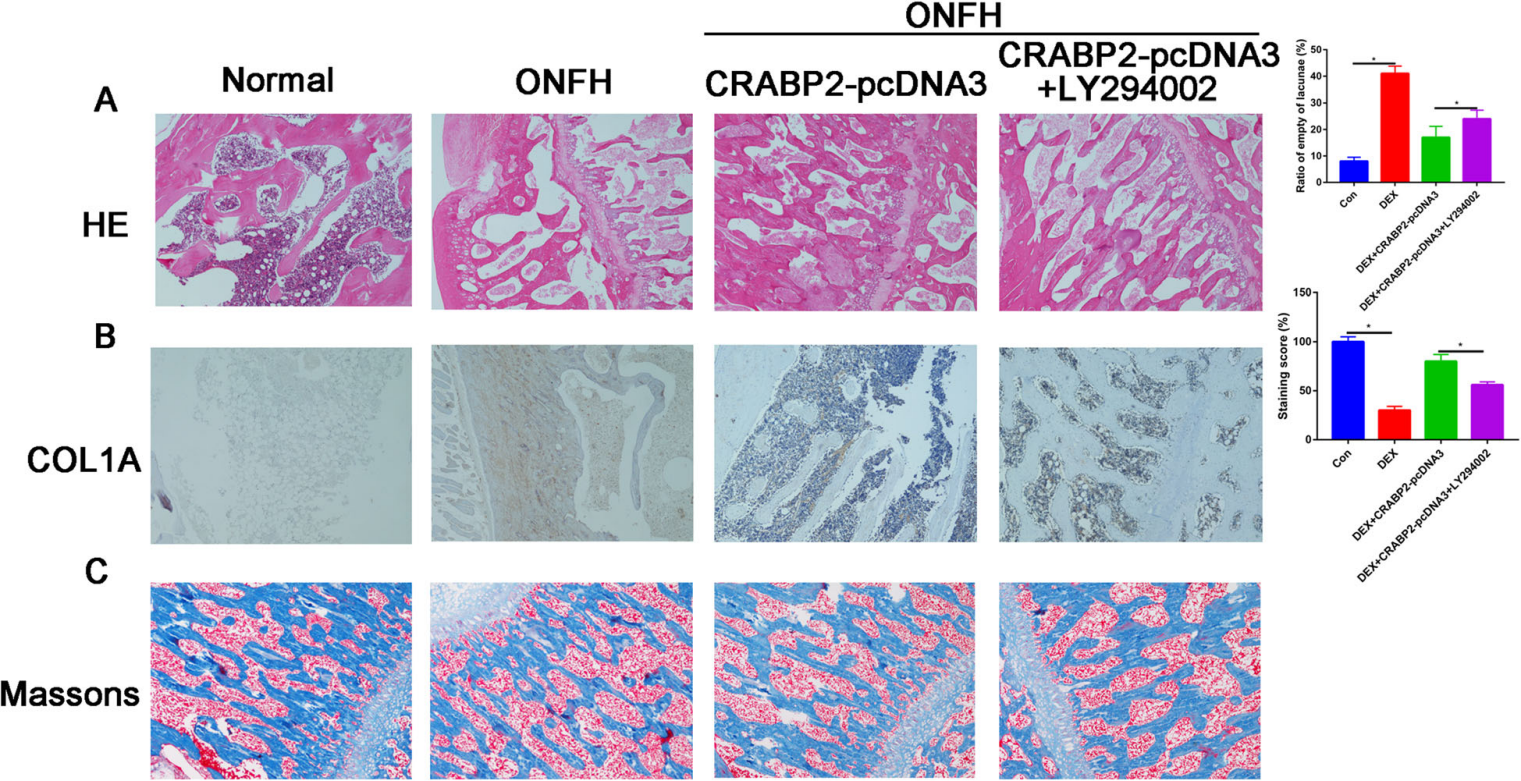
The effects of CRABP2 overexpression on delaying progression in ONFH animal model. **a** HE staining was measured histological changes in the trabecular bone microstructure after administration with CRABP2 overexpression plasmid. **b** Immunohistochemistry results of the COL1A expression in femoral head after administration with CRABP2 overexpression plasmid. **c** Masson staining results in each group (×100 magnification)

HE staining showed that the bone trabecula was thinner, sparser, smaller, and more commonly fractured and disordered in the ONFH group compared with the control group. Administration with overexpression of CRABP2 could prevent the trabecular bone destruction and formulation of fat vacuoles (Fig. 4). What is more, treatment with overexpression of CRABP2 plasmid was able to reverse downregulation COL1A protein expression in DEX-induced animal model (Fig. 4). Masson’s trichrome staining results demonstrated that the content of collagen fibers in ONFH group was obviously less than the control group and overexpression of CRABP2 could partially reverse the inhibitory of DEX on the collagen fibers. These promotion effects were reversed, in part, when LY294002 was added.

## Discussion

The causes of ONFH mainly include hormones, alcohol abuse, hip trauma, and high-dose or abnormal use of glucocorticoids administration. Previous studies have found that DEX-evoked oxidative stress accelerates apoptosis of osteoblasts, leading to the suppression of osteoblastic proliferation and finally lead to ONFH. The mechanisms underlying DEX-induced ONFH remain unclear.

The strength of this study lies in being the first to examine the association between the CRABP2 and ONFH in vivo and in vitro. Another strength of the current study is that overexpression of CRABP2 activated the PI3K/Akt signaling pathway and finally delayed the progression of ONFH. Finally, we performed integrative analysis by combining bioinformatics analysis with experiment validation to understand the mechanism of CRABP2 acting on ONFH.

This is the first integrated study of the role of CRABP2 in the progression of ONFH. In this study, we firstly performed bioinformatic analysis of GSE10311 and identified that CRABP2 as the core gene in DEX-induced apoptosis of osteoblast.

Osteoblasts were treated with various doses of dexamethasone (DEX; 10^-8^ to 10^-4^ M) for 24 h. DEX reduced the number of osteoblast in a concentration-dependent manner. Finally, we choose 10^-4^ M DEX for further study. Annexin-V/PI double staining and DAPI staining assays were used to detect apoptosis. DEX significantly increased the apoptotic cells. Moreover, after DEX treatment, the expression of apoptotic markers (Cleaved caspase-3, Caspase-3, and Bax) was increased, and the expression of an inhibitor of apoptotic proteins (Bcl-2) was decreased.

While overexpression of CRABP2 could partially reversed the pro-apoptotic effect of DEX. Previous studies have identified the biological function of CRABP2 in the metastasis of breast cancer [22] and lung cancer [15]. CRABP2-pcDNA was used to increase the expression of CRABP2 and elucidate the underlying mechanism of CRABP2. CRABP2 is a member of the retinoic acid (RA) and lipocalin/cytosolic fatty-acid binding protein family and plays an important role in maintaining the cell viability. Wang et al. [23] also identified that CRABP2 could significantly promote osteogenesis of C2C12 cells. The apoptotic effects of CRABP2 overexpression were attenuated by blocking with a PI3K inhibitor (LY294002). Previous studies have found that activation of PI3K/Akt signaling pathway in cells can maintain cell survival through inhibition of apoptosis by targeting Bas and caspase-9. We also found that PI3K/AKT signaling pathway was suppressed by DEX treatment. Overexpression of CRABP2 activated phosphorylation of PI3K/Akt signaling pathway. Chen et al. [16] revealed that CRABP2 knockdown suppressed tumor growth in nude mice xenografts.

In the present studies, we found that CRABP2 overexpression reduced DEX-stimulated bone loss in trabecular bone, which identified by HE and Masson staining.

Nevertheless, our study is not without limitations. First, the downstream mechanism of CRABP2 is complex; hence, future studies should focus on the downstream pathways. Second, this study does not actually illustrate the interaction between CRABP2 and other proteins. This issue is a limitation of our study and we will explore the interaction domain of CRABP2 and other proteins in future investigations.

## Conclusion

In conclusion, we first conducted a bioinformatic analysis of the DEGs of control and DEX-treated human osteoblasts. We determined that CRABP2 overexpression could reverse DEX-induced apoptosis in osteoblast through the PI3K/Akt signaling pathway. These outcomes indicate the value of CRABP2 as a potential therapeutic target for ONFH.

## Data Availability

We state that the data will not be shared since all the raw data are present in the figures included in the article.
